# Lessons Learned in the Design and Implementation of Virtual Telemedicine Curriculum for Third Year Medical Students Incorporating New AAMC Telehealth Competencies

**DOI:** 10.15694/mep.2021.000154.1

**Published:** 2021-06-04

**Authors:** Matthew P. Abrams, Ethan Hartman, Denise Kay, Christine Kauffman, Analia Castiglioni

**Affiliations:** 1 University of Central Florida College of Medicine

**Keywords:** Telehealth, Covid-19, Telemedicine, Transition, Clerkships

## Abstract

This article was migrated. The article was marked as recommended.

**Background:** Telehealth, including Telemedicine, is the use of electronic communications technology to provide healthcare at a distance. There is a growing need to train future physicians to be adept and knowledgeable of telehealth. The Association of American Medical Colleges (AAMC) recently defined six core competency domains for Telehealth for medical residents and attending physicians.

**Methods:** A multidisciplinary team of medical educators, Telemedicine practitioners, instructional technology experts and a senior medical student designed a Telemedicine curriculum centered on five primary educational activities. Training moved progressively from novice Telemedicine experiences to Telemedicine encounters with standardized patients and post-encounter debriefs to promote active learning, engagement, and self-regulation. The Telemedicine curriculum was prioritized and delivered to the entire class of 2022 (114 third-year medical students).

**Results:** Student satisfaction surveys and post formative quiz items were used to assess the impact of the Telemedicine Curriculum. Over 95% of surveyed students agreed or strongly agreed the course was organized and helpful in developing clinical skills in preparation for clerkship. Students particularly valued the opportunity to engage in patient encounters while learning Telemedicine-specific patient safety and communication skills.

**Conclusion:** With careful attention to instructional design, active learning formats that are historically successful in face-to-face settings can be equally successful in virtual settings. Standardized patients and peers can be trained to provide appropriate feedback in the right virtual setting.

## Background

Telehealth is defined as the use of electronic communications technology to provide care at a distance ( Association of American Medical Colleges, 2020c).While telehealth is often used interchangeably with the term “Telemedicine”, the latter is considered a specific form of telehealth regarding the use of clinical and medical information that is exchanged from one site to another through electronic communications to improve patient health ( Penchansky and Thomas, 1981; Tuckson, Edmunds and Hodgkins, 2017; American Academy of Family Physicians, 2020; Centers for Disease Control and Prevention, 2020; Teoli and Aeddula, 2020). Although telehealth is associated with reduced healthcare costs, decreased wait times, and improved access to care, the adoption of telehealth in clinical practice has been slow due to concerns over privacy, reimbursements, and limited access to technology ( Abrams, Elsner and Burrill, 2018; American Well, 2019). Furthermore, medical schools have struggled to find time and space in the curriculum to introduce Telemedicine competencies sufficiently.

The COVID-19 pandemic has exacerbated issues of access to care across the United States, increasing the need for telehealth services (Centers of Disease Control and Prevention; Boulware, 2020; Assistant Secretary for Planning and Evaluation, 2020). By April of 2020, telemedicine visits in the US had increased from 0.1% to 43.5% of all healthcare visits and the percentage of patients using Telemedicine services is projected to level off at approximately 21% after the pandemic ( Assistant Secretary for Planning and Evaluation, 2020). As telehealth visits become integral to the modern practice of medicine, it is critical that medical schools prepare learners to deliver virtual patient care.

As part of its New and Emerging Areas in Medicine Series, the Association of American Medical Colleges (AAMC) recently defined six telehealth core competency domains for medical residents and attending physicians: 1) Patient safety and appropriate use of telehealth 2) Data collection and assessment via telehealth 3) Communication via telehealth 4) Ethical practices and legal requirements for telehealth 5) Technology for telehealth and 6) Access and equity in telehealth ( Association of American Medical Colleges, 2020c). These competencies span the continuum of medical education across three levels: 1) Entering Residency (undergraduate medical education), 2) Entering Practice (graduate medical education) and 3) Experienced Faculty Physician (continued medical education). Literature is scarce regarding how to best incorporate these core competency domains in an undergraduate medical education curriculum.

Despite modern medical students’ familiarity with technology ( Pathipati, Azad and Jethwani, 2016; Sharma *et al.,* 2019; Association of American Medical Colleges, 2020b), these skills do not always translate to use of technology in patient care. Formal training is still required to ensure standard of care and protection of patient privacy in Telemedicine-based patient encounters.

In early 2019, less than half of medical schools in the United States had an established pre-clerkship telehealth curriculum ( Waseh and Dicker, 2019). Nonetheless, students, residents and providers are increasingly required to use technology to meet the needs of their patients. Urgent need for Telemedicine services due to COVID-19, mandates for physical distancing, decreased access to clinical rotations for medical students ( Waseh and Dicker, 2019; Association of American Medical Colleges, 2020a) and a shift to virtual education, served as an opportunity to incorporate new content into an overloaded curriculum previously unable to accommodate telehealth education. In this innovations article, we describe a curriculum rapidly designed by a multi-disciplinary team at the University of Central Florida College of Medicine (UCF COM) to introduce the new AAMC core competency domains for telehealth in the wake of the COVID-19 pandemic.

## Context

UCF College of Medicine (COM) MD curriculum is structured following a two-year pre-clinical and two-year clinical training model. At the beginning of the COVID-19 pandemic, the students in the class of 2022 were preparing to leave the pre-clinical portion of the MD program and enter the clinical clerkships. However, the need for social distancing, increase in hospital census, and shortages of personal protective equipment (PPE) resulted in widespread delays in students’ access to clinical rotations across US medical schools. This created new challenges and opportunities for curricular change, resulting in a 12-week “transition to clerkships” module, an integrated multi-specialty curriculum intended to prepare students for clerkship responsibilities.

The opportunity to teach students new skills as part of the transition to clerkship course aligned with the mandated format of online instruction. The module was designed through collaboration between faculty (medical educators, educational psychologist, and practicing Telemedicine providers), instructional technologist, and a senior medical student. The module curriculum included synchronous and asynchronous multi-media material, simulations, and standardized patient (SP) interactions. The course was delivered to 114 third-year medical students over five sessions during the first six weeks of the Transition to Clerkship course (Supplementary File 1). Implementation of the course relied heavily on UCF COM resources, such as the Clinical Skills and Simulation Center staff, standardized patient program, course coordinators and instructional technologists.

## Methods

UCF COM utilizes active learning modalities to promote engagement and self-regulation in learning. The goal was to design the Telemedicine Course to efficiently introduce the new AAMC Telehealth Core Competencies while also providing students with a safe environment to apply knowledge and practice skills. Although all Core Competencies were integrated, the interventions focused on competency domains 1-3 (establishing the virtual relationship, digital communication, “webside” manner, and the virtual physical examination), as they best aligned with the level of learner and available resources. Since this curriculum was designed for rising clerkship students, the competencies introduced and elaborated in the course were at the level of “Entering Residency.”

The initial step was to identify course objectives that would integrate the telemedicine learning goals with existing curricular (See Table 1).

**Table 1.** Telemedicine Course Objectives

Demonstrate basic understanding of Telehealth and Telemedicine modalities, clinical applications, and how they compare to standard healthcare delivery. Understand core competencies for virtual healthcare, to include “webside” manner and virtual physical exam skills. Describe general troubleshooting tips during Telehealth encounters for network, connection and audiovisual concerns. Understand principles of Telemedicine Ethics, Professionalism and “Netiquette”. Discuss ethical principles in practice and apply value clarification when constructing range of preferences for treatment with the patient. Apply knowledge and principles of culture, health and society in patient-centered care. Model optimal medical decision-making and patient engagement by aligning Evidence-Based-Medicine and Shared-Decision-Making to produce ethical patient care outcomes. Demonstrate the skills to safely and effectively engage and document telemedicine encounters. Enhance clinical knowledge and patient care/safety through information retrieval from electronic evidenced-based resources. Identify types of medical errors and determine action plans to correct, mitigate or prevent the errors. Make recommendations to address identified problems using relevant principles from Longitudinal Curricular Themes of Clinical Skills and Reasoning, Culture, Health and Society, Patient Safety, Medical Ethics, Medical Informatics, and Geriatric Care.

UCF COM’s communication and interpersonal skills curriculum follows Kalamazoo’s Essential Elements of Communication (EEC) framework ( Makoul, 2001). UCF COM’s existing behavior-anchored EEC checklist ( Rider and Nawotniak, 2020) was modified to include relevant Telemedicine communication behaviors in alignment with AAMC Telehealth core competency domains. This revised checklist was then abridged to create a usable one-page Observer Checklist ( Table 2). Using the checklist, peer observers and SPs could assess the performance of students and provide feedback in Telemedicine-specific domains.

With the Telemedicine Course Objectives identified, faculty collaborated in designing the learning activities to introduce and reinforce target knowledge, skills, and attitudes throughout the module. The resulting course centered on five primary educational activities (described below) that moved progressively from a novice’ Telemedicine experiences to Telemedicine encounters with standardized patients and post-encounter debriefs.

**Table 2.** Telemedicine SP Encounter Communication Skills - Observer Checklist

1. OPEN THE DISCUSSION (Introduction, Patient Opening, Agenda Setting)			
ESTABLISHING THE VIRTUAL RELATIONSHIP	Does not confirms patient’s full name and date of birth Does not confirm patient’s actual location Does not obtain telehealth informed consent	Confirms patient’s full name and date of birth Confirms patient’s actual location Obtains telehealth informed consent	Verifies state (i.e., location) of patient at time of the visit Verifies that patient is in a safe space to discuss sensitive information
2. BUILD A RELATIONSHIP (Listening, Empathy & Attitude, Non-verbal behavior)			
DIGITAL COMMUNICATION AND WEBSIDE MANNER	Spoke quickly and used colloquial non-professional terms Poor eye contact, poor lighting Not animated, looked bored Tele-office was loud, or distracting in appearance Informal attire suggestive of FaceTime with family Unable to help the patient	Sounded professional but occasionally tone was informal Adequate lighting, good camera placement; looked away at times for unclear reasons Uses hands and face gestures for emphasis Appropriate attire Asked patient to center themselves in field for better lighting, etc.	Appropriate language, excellent projection and use of microphone Looked at camera and explained why he looked away if needed Used hands and face to explain, or demonstrate Office was tidy without distractions Solid colors with natural tones that do not distract Able to troubleshoot technology and share screens as needed
3. GATHER INFORMATION (Context, Questions, Organization& transitions, Personal Privacy)			
THE VIRTUAL PHYSICAL EXAM	Does not describes physical exam observations Does not mention surrounding’s environment Does not verbalize instructions for you to assist with the physical exam Does not set expectations regarding the virtual physical exam Does not mention limitations to virtual exam	Describes some physical exam observations Makes mention of surroundings environment Verbalizes instructions for you to assist with the physical exam Sets expectations regarding the virtual physical exam Mentions that there are limitations to virtual exam	Makes mention of surrounding environment privacy and safety Well demonstrated instructions for you to assist with the exam Sets clear expectations regarding the virtual physical exam Notes abnormality and requests in-person exam or testing
4. UNDERSTANDING THE PATIENT’S PERSPECTIVE (Patient concerns, patient beliefs and preferences, Expression of feelings)			
5. SHARE INFORMATION (Vocabulary, Patient understanding of illness, Clinician information and explanation)			
6. REACH AGREEMENT- Planning Evaluation and Treatment (Negotiation, Implementation)			
7. PROVIDE CLOSURE (Patient next steps, Physician conclusion)			

* Adapted from University of Central Florida COM Essential Elements of Communication Checklist

## Educational Activities

Students participated in five activities over five weeks, with students self-selecting into groups of six. Average student preparation and participation time ranged between 2-4 hours per week (see Supplementary File 1 for comparative summary). Descriptions of each activity are provided below.

### Educational Activity 1: Virtual Interview-Telemedicine Simulation (synchronous)

The Telemedicine course began with a Telemedicine group simulation, the session was a two-part virtual simulation in which students, in groups of three, conducted a virtual interview with Case A: symptoms consistent with Urinary Tract Infection or Case B: symptoms consistent with Diarrhea.

In the first simulation, students engaged in a virtual simulated encounter where they gathered a patient history and communicated clinical impressions and a plan of care to a patient (SP). Between simulation 1 and simulation 2, an experienced Telemedicine practitioner presented Basic Telemedicine Skills. The virtual presentation highlighted and addressed potential misconceptions related to a virtual office visit that students may not have considered, such as the location of the patient and the ability to engage in a private conversation. Students applied these Telemedicine principles in the second simulation encounter (Case A or B, whichever case remained for the students to complete).

After each simulation, students completed a formative quiz addressing various topics related to Telemedicine (e.g., establishing a valid physician-patient relationship, assumptions about HIPAA, confirming patient location, engaging in a private conversation) and other aspects of the case (e.g., relevant medical error, cultural humility, and ethical considerations). In a separate virtual breakout room, faculty had access to students’ quiz responses in real time, allowing them to determine what content needed additional reinforcement during the second debrief. After each quiz, a debrief focused on behaviors consistent with Telemedicine best practices and the clinical case.

### Educational Activity 2: Telemedicine Self-Learning Modules (asynchronous)

A self-learning module (SLM) is an instructional unit with well-defined learning objectives that students can complete independently. Students at UCF COM completed asynchronous SLMs, and video tutorials developed by the multidisciplinary team.

Each SLM covered Telemedicine core competencies, safety, ethics, legal requirements, and etiquette. During and after each self-learning module, students completed formative quizzes that assessed comprehension. The quizzes could be taken multiple times and a passing score of 70% or higher was required to continue the Telemedicine curriculum.

In addition to the SLMs, three asynchronous instructional video examples and tutorials were provided to students. In the first video, an experienced Telemedicine provider and SP demonstrated a typical clinical encounter between physician and patient. In the second video, a senior medical student and SP demonstrated a typical new patient intake from a medical student’s perspective. The senior medical student and SP scripted and performed the third video, demonstrating common mistakes in Telemedicine encounters.

The descriptions of the content and objectives for the second educational activity materials are summarized in Supplementary File 1.

### Educational Activity 3: Self-Reflection and Discussion Board Activity (asynchronous)

The goal of this multicomponent activity was for students to critically evaluate one of their previous in-person simulated clinical encounters to identify how the encounter would be different if it were a Telemedicine encounter. The intention was to reinforce the potential benefits of the encounter taking place in the patient’s natural environment (e.g., patient comfort) versus the potential loss of not having direct physical access to the patient, and for students to recognize the need to assess if they had adequate information to appropriately care for the patient via Telemedicine or if they needed to escalate the level of care to an in-person visit.

In the first reflection activity, students reviewed a video of a clinical skills encounter they completed during their second pre-clinical year. The task for the original encounter was to complete a hypothesis driven history and physical, formulate a differential diagnosis and give their initial impressions to the patient. Students were instructed to review this encounter and to reflect on what they would do differently if the encounter were a Telemedicine encounter. The students were given the following reflection prompts and directed to post it to their group’s peer discussion board:

 According to the information from the Telemedicine SLMs, as well as your experience in the Telemedicine Interprofessional Simulation, what are five things you would do differently if this were a Telemedicine encounter with this patient?

 What are two things you would do the same if this were a Telemedicine encounter with this patient?

 Describe one opportunity that a Telemedicine encounter provides that you would not have if you saw this patient in the clinic

In a second reflection activity, students were tasked with submitting a post with the following prompt:

 If you were seeing this patient today with only the information you could have gathered via Telemedicine, could you successfully complete your assessment or would you have to refer them for an in-person visit today? Justify your answer.

Students were subsequently tasked with reviewing and responding to at least two group members’ posts. Reflection groups comprised the same groups of six students that were split into two groups of three in the first simulation sessions. Students were provided with four reply options aimed at promoting reflection about similarities and differences between their reflections and that of their peers (for peer response prompts see Supplementary File 1, Activity 3).

The goal for the asynchronous posting, reviewing and responding to the discussion board posts was to encourage students to offer a distinct perspective and expand their own thinking through exposure to different perspectives. These reflection activities served as the foundational experiences to prepare students for the subsequent activities wherein Telemedicine skills would be developed and reinforced.

### Educational Activities 4 & 5: Telemedicine Standardized Patient Encounters and Faculty Debrief (synchronous)

In both weeks 4 and 5, paired students (Interviewer and Observer) participated in synchronous Telemedicine SP Encounters where they interviewed a standardized patient remotely (via Zoom) and practiced Telemedicine-specific skills. Students received individual feedback from the patient, the peer observer, and faculty after each patient encounter. Encounter-specific learning objectives were defined by faculty (see Supplementary File 1: Objectives row) reflecting the content that had been emphasized in Telemedicine educational activities 1-3 and pre-clerkship curricular modules. Case scenario complexity was designed to increase from encounter I (week 4) to encounter II (week 5).

The two Telemedicine SP encounters were structured in a similar fashion, consisting of four sequential virtual components: 1) encounter pre-brief, 2) SP encounter, 3) patient feedback and 4) small group faculty debrief (see Supplementary File 1: SP content description). Ten virtual patient encounters took place simultaneously via zoom, for a total of 10 student-pairs. Students were moved through each virtual activity by the zoom session host.

Pre-brief: Students were first virtually briefed as a group and instructions were reviewed, including session learning objectives, timing of each component, and anticipated flow on zoom (e.g., moving from virtual patient room to virtual debrief room, expected announcements from the host, etc.). At this time, students were presented with the age, gender, chief complaint and vital signs of the patient they were about to see, as well as their individual tasks.

SP encounter: The student interviewer was allotted 15 minutes to obtain a symptom-based history and share their clinical impression and plan. With their camera and microphone off, peer observer students took notes as the interview progressed, using the Observer Checklist ( Table 2) as a guide to later share observations and feedback during the debrief.

Encounter I presented a common clinical presentation (insomnia) for the students to practice communication skills (including the new Telemedicine domains), history taking and clinical reasoning. Encounter II presented a more complex case scenario with the role of students switching from interviewer to observer and vice versa. In addition to the skills practiced the week prior, this encounter required students to engage in more patient education and shared decision making through Telemedicine.

SP Feedback: Following the encounter *,* the SP utilized the same Observer Checklist to provide feedback to the interviewer student from a patient’s perspective.

Debrief: Once SP feedback was completed; student pairs were moved to a virtual breakout room for a 30-minute small group debrief with peers and a faculty member (20 students + faculty facilitator). Faculty provided a psychologically safe environment, offered feedback, and promoted self-reflection. The debrief allowed observer students to share their observations and for the group to highlight Telemedicine skills and challenges. Faculty closed the session with ultimate lessons learned and take-aways from the session.

## Impact

The new Telemedicine course successfully introduced students to the new AAMC Telehealth Core Competencies over five weeks in a time of urgent need for online medical education.

We utilized two data sources as indicators of curricular success, including 1) student satisfaction surveys and 2) post formative quiz items from educational activity one.

The anonymous satisfaction surveys provided students a space to provide narrative feedback and were collected regarding the SP encounters and the overall Transitions to Clerkship course. Relevant data was redacted and used in this educational report.

All 114 students were sent an 11-item survey following each of the two SP encounters and faculty debriefs (Post-SP Encounter Survey 1 and 2; Appendix 1). Response rate was 45% (51) for post-SP encounter survey 1 and 31% (35) for post-SP encounter survey 2. All students completed the Transitions to Clerkship course evaluation.

### Students Satisfaction Surveys and the Educational Process

Student satisfaction surveys were reviewed to inform course designers about the educational process of the Telemedicine Course overall, as well as students’ report of knowledge or skill acquisition as a result of the course. Overall, the impact of the curriculum was overwhelmingly positive. Both students and faculty appreciated the online format, with nearly unanimous support and approval for the new curriculum. Students felt that this course prepared them appropriately for Telemedicine encounters and helped them prepare for third year clerkships.

Students reported that it was meaningful to practice assessing unseen physical aspects of the patient in a way that maintained rapport (e.g., assessing if a patient was in a wheelchair while their legs were offscreen, noting the patient home setting etc.) and getting the patient to adjust the lighting, sound, or camera as appropriate for proper virtual interaction. Students appreciated learning about the differences between Telemedicine and in-person encounters, even wishing to have more practice with Telemedicine to improve their deficits in the virtual setting. Themes identified related to the educational process are described below.

Virtual SP Encounters as a Valuable Learning Tool: Student feedback was exceedingly positive, suggesting that the Telemedicine course was well-received and provided students with tangible Telemedicine skills and useful, new telehealth knowledge. Students were generally very satisfied with the organization of the course and communications from faculty and SPs.

The surveys assessed students’ level of satisfaction with feedback received from SP, feedback provided during the debrief, and overall Telemedicine encounter experience. Over 95% reported they were either moderately satisfied, quite satisfied, or extremely satisfied with the Telemedicine encounter experience in each survey. Quantitative and qualitative data from both post-SP encounter surveys revealed that the SP encounters were well-received and that most students found them helpful (Appendix 1, Post-SP Encounter Survey 2).

Positive Interactions and Organization: Students had positive interactions with SPs and faculty via virtual communications. One student wrote, *“I think it was helpful to simulate a Telemedicine visit. It was good experience to have to navigate some of the challenges of not being present with the patient when conducting the interview and considering appropriate management.”* Regarding the debrief, a student commented *, “The debrief [allowed] a great balance between discussing the clinical aspect of the case and the Telemedicine skills involved with the case.”*

Greater than 94% of students agreed or strongly agreed the Telemedicine encounter experience was well organized. Narrative comments reflected the students’ general positivity toward the planning and organization, and appreciation for the clear explanation of Zoom logistics. Most comments focused on the smoothness of transitions, the coordination of the breakout rooms, and the clearness of instructions.

Appreciation for Patient Encounters During Remote Instruction: Students generally expressed gratitude for the utility and timeliness of this course. When provided the opportunity to provide narrative feedback at the end of the 12-week Transition to Clerkship course, one student shared *“I think one of the best series of sessions was our Telemedicine training. Due to the nature of Telemedicine, I think that online delivery is actually the optimal presentation of this part of our curriculum.”*

Throughout the five-week Telemedicine course, learners and educators alike expressed that they felt the online delivery of information was appropriate and effective for learning. For example, one student shared, *“A big part of Telemedicine is technological trouble shooting and being flexible with your practice. Learning how to perform Telemedicine encounters in a pandemic setting is the closest thing we can do to simulate real life as many providers have had to genuinely make that transition. I feel prepared in case of future emergency and confident that I can adapt to still serve my patients.”*

## Lessons Learned

A Telemedicine curriculum can be rapidly developed to meet the needs of students transitioning to clerkships. Online Telemedicine education is a valuable tool that can save time, money, and effort among students, medical educators and eventually patients. Such a curriculum can be utilized longitudinally for re-use in various clinical scenarios and can be tailored to suit many educational contexts. Although it is feasible to create, those wishing to design and deliver a similar curriculum may benefit from these lessons learned regarding the design, resources needed, implementation, pedagogical approach, and evaluation of this course.

### 1. Careful selection of a multidisciplinary team that represents all stakeholders

An advantage in the creation of this Telemedicine course was the collaboration of a multidisciplinary team. It is recommended to include a diverse cohort with carefully selected stakeholders in the development of a Telemedicine course, including but not limited to clinical faculty, Telemedicine practitioners, experts in medical education, and medical students. For example, student feedback demonstrated the importance of optimizing the curriculum development team to include clinician educators with expertise.

### 2. Careful selection of the correct resources, content, and pedagogical approach

Developers of a successful Telemedicine course must ensure students and faculty have a stable internet connection, audiovisual communication technology, and standardized patients who can be trained to interact online. Subscribing to an appropriate interfacing platform (e.g., Zoom, Skype etc.) is also crucial for ensuring connection between students, faculty, and standardized patients. Content experts are highly recommended for the creation of SLMs, using the literature to develop the most up to date guidelines.

Based on surveys and quizzes from students in this Telemedicine course, the multiple pedagogical approaches that were historically used in face-to-face sessions translated equally well to the online format. Students perceived these approaches as just as effective if not more effective in the virtual setting. We believe this was, in part, due to the diverse team of faculty that operated from a shared mental model for how to prioritize content and pedagogy selection. The team should consider questions such as “What is essential to meeting the learner in the Zone of Proximal Development?” versus “What would be nice to have?” and “How can we trim down content and translate expert knowledge to understandable content for learners at this level?”. We found the diversity of team members and having a way to prioritize content and pedagogical decisions contributed favorably to the richness of discussion while designing a course that led to positive learning outcomes.

### 3. Utilize progressive learning formats

The learning progressions started with a novice’ Telemedicine simulation experience, followed by a formal introduction to Telemedicine principles, subsequent opportunities for reflection on what students would do differently if prior encounters were a Telemedicine encounter, ending with the opportunity to practice Telemedicine skills in groups and then, individually with standardized patients. Rather than a simple didactic session, this progressive approach allowed students to practice what they learned repeatedly in a stepwise fashion. Spreading the information across five weeks appeared to provide enough time for students to digest the material and adopt the skills.

### 4. The value of SP Feedback in face-to-face simulations translates to virtual simulations

SP Feedback in face-to-face instruction is equally useful in virtual simulations if SPs are provided with the training to use this technology and in how to give feedback in the virtual setting. Sample comments from student’s narrative feedback highlight the important contribution of SP feedback in this learning experience. For example, consider this student comment from education activity 1, where SP feedback wasn’t provided - *“Great use of standardized patients. If possible, allow some time for the SPs to give us feedback about our rapport and ‘webside’ manner; they’re really good at giving this type of feedback.”*

### 5. Optimize student autonomy when possible

Recognizing the importance of social interaction and the benefits of peer interaction for promoting learning, UCF frequently utilizes small groups as an integral part of the learning environment. Given the potential social isolation students might experience with reliance on virtual learning, the students were given the opportunity to self-select into their own groups of six. In this way, students could work with peers they had at least some familiarity with, if not well-established connections. Students did report feeling more comfortable and we found that providing students the autonomy to choose their own groups addressed two problems. First, it promoted virtual socialization during a time of heightened social isolation. Secondly, as many students returned home during the pandemic, they were in different time zones which influenced their availability and partially influenced who they chose to be in their group. With limited information on students’ location, it would have been difficult for faculty to coordinate simulation rotation schedules. Allowing students to select who they wanted to work with allowed schedules to better align.

### 6. Value of observing a peer in Virtual Telemedicine Simulation

Previous literature supports the value of observing peers or role models in clinical settings( Horsburgh and Ippolito, 2018) and the value of having an observer appeared to translate to this simulated clinical encounter. For example, one student shared that *“the inclusion of an observer was really valuable for both the observer and the interviewer.”* Similarly, educators’ impressions of the impact on learning when the students switched roles were positive. Students shared with faculty that, by observing a similar encounter with the observer checklist they had a deeper appreciation for the difficulties of effectively accomplishing some of the checklist items than when just conducting the simulation interview alone. Moreover, students benefitted from simply seeing how a peer approached a similar clinical situation in their own personalized way. Particularly for Educational Activity 5 (SP encounter II), students appeared to value observing a peer educate their patient about the pitfalls of taking antibiotics when not clinically indicated. As one student shared, *“I think it was a great teaching moment to have the privilege to observe.”*

### Faculty Debrief and time constraints

The same challenges typically found in face-to-face group debriefs were encountered in this curriculum’s virtual debriefs. Students found it difficult to engage in a two-way conversation using a zoom format and faculty shared they experienced similar limitations with providing individualized feedback over zoom in a larger group format. No agreement could be reached about the appropriate length of time for faculty debriefs.

Timing was an issue for some attendees of this Telemedicine course, as students asked for more time interviewing the standardized patient and less time in lectures. One student commented “ *would have benefitted more from more interview time with the patient (no one was able to do a full interview) instead of an entire half hour debriefing“.* This desire from students to spend more time in deliberate practice over debrief is important to consider. Although debrief is where most learning is thought to occur, the utility of deliberate practice cannot be underestimated. Another student shared that *“everything was organized, and patient and* *faculty debriefs were helpful”* demonstrating it is also a challenge for educators to balance the different learning styles and preferences of all learners in each cohort. Therefore, designers of a Telemedicine course should strive at least to allow for completion of simulated medical interviews and create an efficient debrief that can be delivered quickly.

Overall time allotted for this course was limited. This five-week Telemedicine course was considered valuable by many students in that it provided the only clinical (albeit simulated) experiences they had during the 12-week transition to clerkship module. Students asked for more time practicing these skills. Although the Telemedicine curriculum was provided to third year medical students, it may be easily adapted across all four years of medical school in a longitudinal and integrated curriculum.

## Conclusion

A new and rapidly assembled Telemedicine course was successfully integrated into the medical school curriculum via virtual didactic and experiential learning. The Telemedicine course was developed in response to the COVID-19 pandemic, which created a need for increased online delivery of medical school education, and more specifically, education in Telemedicine. The developers addressed all six AAMC Telehealth Core Competency domains throughout the course, but the activity design focused primarily on the first three domains as these domains were most applicable for the level of learners and available time and resources. The lessons learned in the design, implementation, and evaluation of this course can be summarized by the fact that with the right resources, people, and pedagogical approach, a Telemedicine curriculum can be rapidly developed. With careful attention to instructional design, active learning formats, historically successful in face-to-face settings, can be equally successful in virtual settings. With the contributions of a multi-disciplinary team, strong support of instructional technologists and a resilient clinical skills staff, we were able to quickly redesign and deploy an integrated course that responded to the need for increased Telemedicine competence. The Web-based conference platform (i.e., Zoom) was a functional platform for simulated telehealth patient visits. We recognize that we may have more resources than other medical schools, but we utilized what we had available to design learning encounters that met a variety of learning objectives in ways that optimized student autonomy, self-regulation and reflection. This curriculum is adaptable to other years of medical training or other healthcare professional programs and it may also be suitable for expansion into a longitudinal curriculum that can be integrated across the medical education curriculum. This robust Telemedicine curriculum can be repurposed to optimize other clinical educators time, money, resources, and effort.

## Take Home Messages

• With the right resources, people, and pedagogical approach, a Telemedicine course with virtual didactics and experiential learning can be rapidly developed in response to the COVID-19 pandemic, which created a need for increased online delivery of medical school education, and more specifically, education in Telemedicine.
• The developers addressed all six AAMC Telehealth Core Competency domains throughout the course, but the activity design focused primarily on the first three domains as these domains were most applicable for the level of learners and available time and resources.
• With careful attention to instructional design, active learning formats, historically successful inface-to-face settings, can be equally successful in virtual settings.
• With the contributions of a multi-disciplinary team, strong support of instructional technologists and a resilient clinical skills staff, an integrated course can be quickly designed and employed to meet the need for increased Telemedicine competence.
• This curriculum is adaptable to other years of medical training or other healthcare professional programs and it may also be suitable for expansion into a longitudinal curriculum that can be integrated across the medical education curriculum; this robust Telemedicine curriculum can be repurposed to optimize other clinical educators time, money, resources, and effort.

## Notes On Contributors

Matthew P. Abrams, B.A., is a third-year medical student at the University of Central Florida’s College of Medicine in Orlando, Florida. His interests include medical education, health equity, psychiatry, global health and student wellness. ORCID: https://orcid.org/0000-0001-6059-4857

Ethan Hartman, B.A., is a fourth-year medical student at the University of Central Florida’s College of Medicine in Orlando, Florida. His interests include medical education, immersive simulation, community service, emergency medicine, and global health.

Denise Kay, Ph.D., MA is an Assistant Professor of Medical Education at University of Central Florida College of Medicine in Orlando, Fl. As a Counseling and Educational Psychologist, her focus is on curricular efficiency, effectiveness and the critical role of active student engagement in learning inmedical education. ORCID: https://orcid.org/0000-0002-1216-9220

Christine Kauffman, M.D. is an associate professor of pediatrics at the University of Central Florida College of Medicine. She is the director of the first-year clinical skills and the second-year gastrointestinal and renal systems courses. Her interests include clinical skills training and the effects of attendance on performance. ORCID: https://orcid.org/0000-0002-2496-1987

Analia Castiglioni, MD., is a Professor of Medicine at the University of Central Florida College of Medicine with over 20 years of experience as a clinician, educator and researcher across the medical education continuum. She has received national and international recognition for expertise in the field, innovative curriculum and medical education scholarship. ORCID: https://orcid.org/0000-0002-8610-4446

## Figures and Tables

**Appendix 1:** Post-SP Encounter Survey 1

**Question**	Strongly Agree	Agree	Neither	Disagree	Strongly Disagree
The content was clearly aligned with the learning objectives.	27 (52.94)	23 (45.1)	1 (1.96)	0	0
The instructions were clear and easy to follow.	31 (60.78)	15 (29.41)	5 (9.8)	0	0
The session was well organized and presented in a logical sequence.	33 (64.71)	15 (29.41)	3 (5.88)	0	0
The briefing was helpful.	28 (54.9)	14 (27.45)	4 (7.84)	5 (9.8)	0
The logistics and notifications were well handled by staff.	20 (39.22)	27 (52.94)	4 (7.84)	0	0
The faculty debrief was relevant and valuable.	24 (47.06)	20 (39.22)	4 (7.84)	3 (5.88)	0
The session was helpful to develop your clinical skills.	24 (47.06)	21 (41.18)	2 (3.92)	1 (1.96)	3 (5.88)
The session prepared you to move on to clerkship rotations.	16 (31.37)	19 (37.25)	11 (21.57)	4 (7.84)	1 (1.96)

	Extremely Satisfied	Quite Satisfied	Moderately Satisfied	Slightly Satisfied	Not at all satisfied
Feedback received from your patient	11 (21.57)	23 (45.1)	14 (27.45)	2 (3.92)	0
Feedback provided during the debrief	10 (19.61)	20 (39.22)	17 (33.33)	3 (5.88)	0
Your overall telemedicine encounter experience	7 (13.73)	18 (35.29)	24 (47.06)	1 (1.96)	0

**Appendix 2:** Post-SP Encounter Survey 2

**Question**	Strongly Agree	Agree	Neither	Disagree	Strongly Disagree
The content was clearly aligned with the learning objectives.	25 (71.42)	10 (28.57)	0	0	0
The instructions were clear and easy to follow.	27 (77.14)	8 (22.86)	0	0	0
The session was well organized and presented in a logical sequence.	26 (74.29)	9 (25.71)	0	0	0
The briefing was helpful.	25 (71.42)	6 (17.14)	1 (2.86)	2 (5.71)	1 (2.86)
The logistics and notifications were well handled by staff.	22 (62.86)	13 (37.14)	0	0	0
The faculty debrief was relevant and valuable.	22 (62.86)	11 (31.43)	1 (2.86)	0	1 (2.86)
The session was helpful to develop your clinical skills.	22 (62.86)	12 (34.29)	1 (2.86)	0	0
The session prepared you to move on to clerkship rotations.	15 (42.89)	17 (48.57)	2 (5.71)	1 (2.86)	0

	Extremely Satisfied	Quite Satisfied	Moderately Satisfied	Slightly Satisfied	Not at all satisfied
Feedback received from your patient	12 (34.29)	21 (60.00)	2 (5.71)	0	0
Feedback provided during the debrief	14 (27.45)	18 (51.43)	2 (5.71)	0	1 (2.86)
Your overall telemedicine encounter experience	17 (48.57)	17 (48.57)	0	1 (2.86)	0
