# Supplementary material for: Lessons Learned in the Design and Implementation of Virtual Telemedicine Curriculum for Third Year Medical Students Incorporating New AAMC Telehealth Competencies
Source: MedEdPublish (2016). 2021 Jun 4;10:154. [Version 1] doi: 10.15694/mep.2021.000154.1 (PMC10939562; doi:10.15694/mep.2021.000154.1)
Supplement: Peer review history

# Reviewer report: Reviewer response for version 1

**DOI:** 10.21956/mep.18976.r27183

**Published:** 2021-07-23

**Authors:** Ben Canny

**Affiliations:** 1 Monash University

This review has been migrated. The reviewer awarded 5 stars out of 5 This article is a very important addition to a sparse literature regarding the teaching of Telemedicine/health, an increasingly important competency. The article sets out well the learning objectives and structure of the individual learning sessions. I particularly appreciated the efforts to which the authors and curriculum designers went to enable contextualisation of the learning opportunities to real world situations. I would have appreciated further advice about the use of specific platforms to support the program. The evaluation was very good, especially the lessons learned; additional information or assessment on student learning would also be helpful.

Reviewer Expertise:

NA

No decision status is available

# Reviewer report: Reviewer response for version 1

**DOI:** 10.21956/mep.18976.r27184

**Published:** 2021-06-21

**Authors:** John Sandars

**Affiliations:** 1 Edge Hill University

This review has been migrated. The reviewer awarded 5 stars out of 5 This is an excellent article that describes the evaluation of a telehealth curriculum. There is a clear description (and justfication) of the curriculum and a clear evaluation of the intervention, including the impact and lessons learned.

Reviewer Expertise:

NA

No decision status is available
